# Evaluation of Workpiece Temperature during Drilling of GLARE Fiber Metal Laminates Using Infrared Techniques: Effect of Cutting Parameters, Fiber Orientation and Spray Mist Application

**DOI:** 10.3390/ma9080622

**Published:** 2016-07-28

**Authors:** Khaled Giasin, Sabino Ayvar-Soberanis

**Affiliations:** 1 Composite Systems Innovation Centre, Department of Mechanical Engineering, University of Sheffield, Sheffield S1 3JD, UK; 2 Advanced Manufacturing Research Centre, Wallis Way, Catcliffe, Rotherham S60 5TZ, UK; s.ayvar@sheffield.ac.uk

**Keywords:** drilling, GLARE, minimum quantity lubrication, infra-red, temperature, fiber metal laminates, coolant, machining

## Abstract

The rise in cutting temperatures during the machining process can influence the final quality of the machined part. The impact of cutting temperatures is more critical when machining composite-metal stacks and fiber metal laminates due to the stacking nature of those hybrids which subjects the composite to heat from direct contact with metallic part of the stack and the evacuated hot chips. In this paper, the workpiece surface temperature of two grades of fiber metal laminates commercially know as GLARE is investigated. An experimental study was carried out using thermocouples and infrared thermography to determine the emissivity of the upper, lower and side surfaces of GLARE laminates. In addition, infrared thermography was used to determine the maximum temperature of the bottom surface of machined holes during drilling GLARE under dry and minimum quantity lubrication (MQL) cooling conditions under different cutting parameters. The results showed that during the machining process, the workpiece surface temperature increased with the increase in feed rate and fiber orientation influenced the developed temperature in the laminate.

## 1. Introduction

Fiber metal laminates (FMLs) are hybrid materials made up of alternating layers of metals and composites bonded together using an adhesive epoxy. Some of the most commonly known fiber metal laminates include Aramid Reinforced Aluminum Laminate (ARALL) and GLass Aluminum Reinforced Epoxy (GLARE). The latter is currently used in parts of the commercial Airbus A380 fuselage structure [1]. The manufacturing and installation of those aerospace structural parts usually require machining operations, such as milling and drilling [1,2]. A limited number of studies have been reported on the machinability of FMLs [3,4,5,6,7,8,9]. Table 1 summarizes some of the most work carried out on drilling those FMLs of which several studies looked into the effect of tool type, size and coating on the hole quality [9,10]. While other studies looked into the impact of coolants, workpiece thickness and fiber orientation on the hole quality [6,7,8,11]. Other studies reported that the thickness of the laminate can influence the heat generation and the milling edge condition in terms of increased delamination, deformation and chip adhesion to the cutting tool [1], while semi-dry milling was found to improve the edge quality when milling thin GLARE laminates [1].

During the machining process, the heat generation occurs due to the continuous friction between the workpiece and the cutting tool. Approximately 98% of the energy in machining is converted into heat [13]. The rise in the temperature can adversely affect the properties of the workpiece and cutting tool materials, induce thermal damage to the workpiece causing dimensional changes, and accelerate tool wear or result in chemical changes in the tool-workpiece materials reducing the quality of the machined parts [14]. Moreover, the rise of cutting temperatures in composites can lead to matrix burnout, acceleration of fiber pull-out and can cause a reduction in the shear strength of the laminate [15]. The use of coolants during the machining process proved to be a powerful method for reducing the associated cutting temperatures. Nevertheless, the impact of machining temperatures remains a major challenge, especially in dry and high-speed machining environments. One of the most important factors which govern the temperature build up during the machining process is the thermal conductivity of the workpiece material [16]. Materials with a low thermal conductivity are subject to higher increase in temperature during machining due to their inability to rapidly dissipate heat. In addition, the alloy content in the material can also affect the machining temperatures, Ozek et al. [17] previously reported that when drilling different aluminum alloys (A5083, A6061, A7075-T651, A1050), the temperature of the workpiece can reach as high as 242 °C in A5083 and 164 °C in A1050 using the same cutting conditions due to the difference in their alloying content. The heat generated when machining composites is distributed differently than when machining metals [18]. In metals, approximately 75% of the heat is generated at the shear zone, 20% at the chip sliding on the tool face and about 5% is produced due to plastic deformation in the metallic workpiece [16] and between 80%–85% of the thermal energy generated is carried away by the evacuated chips [19]. Indeed, the relatively higher ductility of most metals compared to composites means that extensive plastic deformation of the chip takes place during cutting, which increases heat generation and temperature [20]. The drilling of composites is limited by the softening temperature of the matrix system despite fibers having high melting temperatures. Nevertheless, the temperatures generated in the machining process should not exceed the softening temperature of the matrix [21]. In addition, the lack of ductility required for plastic deformation produces very small fragments of chip coupled with a low thermal conductivity of composites, means that a large amount of the heat is dissipated by the workpiece and cutting tool. 

The cutting temperature in drilling is strongly dependent on cutting speed and feed rate [18]. Fliescher et al. [22] previously reported that the workpiece takes a large share of the heat distribution during the drilling process which could be anything between 10%–35% compared to turning and milling operations in which the workpiece takes around 1.1%–20% and 1.3%–25% of the heat, respectively. The rise in the workpiece temperature during the machining process becomes critical when machining composite metal stacks and fiber metal laminates (FMLs) as it could adversely affect the integrity of the composite part in the stack due to poor thermal conductivity in comparison with the metal part, making it more susceptible to thermal damage. Wang et al. [23] reported that the heat loss from the composite metal stack occurs faster when the metal workpiece is placed on the top of the composite workpiece due to better chip evacuation, and the fact that the developed drilling temperatures are higher in the composite than the aluminum due to poor thermal conductivity. Placing the metal workpiece beneath the composite will cause the evacuated metallic chips to be in contact with the composite surface, therefore, increasing the chance for chip clogging and surface damage in the composite part [23]. 

The measurement of the temperature during machining process has been the main objective of many studies in milling and turning operations, and to a less extent in drilling operations especially in composite metal stacks and FMLs. Some of the most common methods for measuring the temperature during drilling process were summarised by Taskesen et al. [24] using either contact methods by embedding thermocouples in the workpiece or the cutting tool, or by using non-contact methods such as infrared pyrometers and infrared cameras. Previous studies on machining aluminum alloys reported that the workpiece temperatures can reach up to 271 °C during dry drilling and up to 440 °C during dry turning [25]. While for composites, the workpiece temperature can be as low as 128 °C and up to 580 °C when drilling composite metal stacks [23,26]. The use of coolants can considerably reduce the machining temperature, previous studies reported that turning Inconel 718 steel using cryogenic and MQL cooling method resulted in drastic reduction in workpiece temperature compared to drilling at room temperature with cryogenic cooling being more effective than MQL [27]. Similar results in temperature reduction were reported when grinding stainless steel 318 using wet and cryogenic cooling [28]. Dhar et al. [29] showed that using MQL when turning AISI 1040 steel resulted in a reduction of 125 °C compared to drilling at room temperature. Shetty et al. [25] reported that using oil–water emulsion, compressed air, and steam reduced the workpiece temperature of an AA6061-15 vol % SiC alloy by 40, 120 and 280 °C, respectively. 

There has been a significant amount of studies which looked into the effect of cutting parameters and coolants on the maximum temperature during machining process for various metals including steel [27,28,29,30,31,32,33,34,35,36,37,38,39], aluminum alloys [17,25,34,40,41,42,43,44], titanium mainly Ti6Al64 alloy [43,45,46] and, to a lesser extent, other material, such as magnesium alloy [47]. While studies on temperature measurements of composite [15,23,48,49,50,51,52,53,54,55,56,57,58,59] and composite metal stacks machining [23,50,60] have surged in the past few years, there have been no reported studies on temperature measurements on machining FMLs. The introduction of heat affected zones into the cutting tool and the edges of the laminate were previously reported to influence the quality of machined FMLs [1] and, therefore, it is considered a particular area of interest, which should be further investigated. In this research, the drilling temperatures of the bottom surface of machined holes in two grades of GLARE fiber metal laminates were compared to determine the impact of fiber orientation. The study also looks into the impact of using minimum quantity lubrication on the developed temperatures compared to dry drilling at room temperature on the exit surface temperature of the laminate. In addition, the study reports the emissivity values of GLARE surfaces, which can be used in other studies that might require temperature analysis using infrared techniques.

## 2. Materials and Methods

### 2.1. Workpiece and Cutting Tool

**Figure 1:**
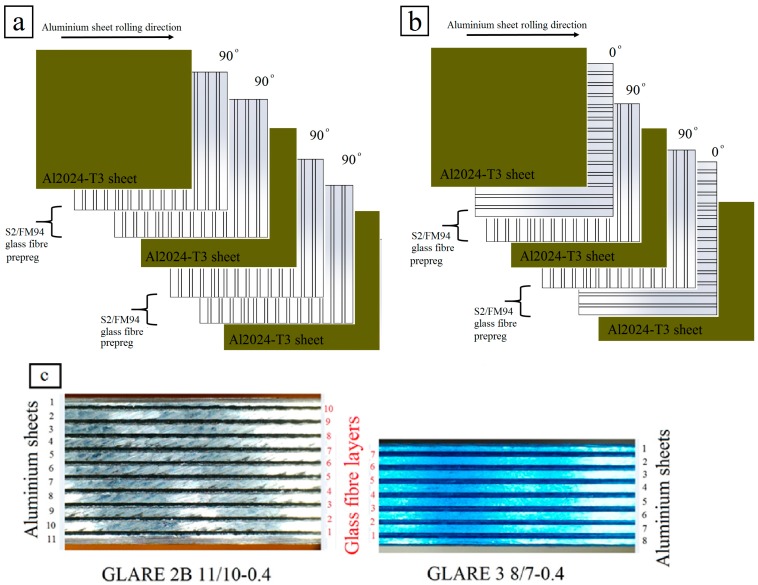
Schematic illustrations of GLARE laminates: (**a**) GLARE 2B with the unidirectional fiber coinciding with the rolling direction; and (**b**) GLARE 3 with the cross-plied fiber layers (**c**) Front view of GLARE 2B 11/10-0.4 and GLARE 3 8/7-0.4 specimens used in the drilling trials [6].

The workpiece materials used in this study were unidirectional GLARE fiber metal laminates. This investigation considered two grades of GLARE panels: GLARE 2B and GLARE 3. Figure 1 illustrates the configuration of GLARE 2B and GLARE 3 laminates. The samples were supplied by the Fiber Metal Laminate Centre (FMLC) in Delft, the Netherlands. Each GLARE sample consisted of layers of Al2024-T3 having a nominal thickness of 0.4064 mm and prepregs of S2-glass fibers embedded in an FM94 adhesive having an approximate thickness of 0.133 mm [1], as shown in Table 2. 

Each fiber layer consisted of two unidirectional prepregs oriented at [90°/90°] in GLARE 2B and [0/90°] in GLARE 3 [6,7,11], where the rolling direction in aluminum sheets is defined as (0°) (see Figure 1). Table 3 depicts some of the mechanical properties of S2-Glass fiber prepreg and Al2024-T3 considered in the experimental work. The samples were cured in an autoclave for around 300 min at elevated temperatures of 120 °C and under a pressure of 6 bars [6,7,11,61]. The aluminum sheets surface are pre-treated and degreased followed by chromic or phosphoric acid anodising and subsequent priming with BR-127 corrosion inhibiting bond primer [1]. The fibers are delivered as a prepreg, including the FM94 adhesive system from Cytec in the UK [1]. The cutting tool considered in this work was OSG HYP-HP-3D ϕ6 mm TiAlN coated carbide twist drills with the point angle of 140° and the helix angle of 30° [6,7,11]. 

### 2.2. Determining the Emissivity of the Surfaces GLARE Fiber Metal Laminate

The emissivity can be described as the amount of thermal energy, which a material radiates in the form of infrared energy, the value of emissivity for materials ranges between 0 (material that does not emit infrared energy) to 1 (black body), which completely radiates thermal energy. Different materials have different emissivities, several factors can affect the emissivity of an object such as its temperatures and the ambient temperature, material size and shape. Since it was previously reported that the heat affected zone at the edges of machined laminates is a phenomenon, which deserves to be considered during machining operations, an attempt is made to evaluate the developed temperature around the hole edges at the exit side of the laminate when the cutting tool is exiting the workpiece. In the current research, the material heating method was used to find the emissivity of GLARE’s upper and lower surfaces and its edges. The outer aluminum sheets of GLARE are usually coated with a thin clad layer to improve surface corrosion resistance; in addition, all aluminum sheets are also sometimes anodized and coated with a corrosion inhibiting primer [1]. The chemical processing of the aluminum sheet gives its surface a varying level of color intensity resulting in a range of emissivity rather than a constant value. The emissivity of GLARE was determined by conducting three emissivity tests, as shown in Table 4. Each test was carried out using different infrared (IR) cameras and different types of coating/tapes, which have known emissivity values. 

There are several methods to determine the emissivity of an object, surface treatment, and material heating are some of the most common and simple methods, the later was used to find the emissivity of GLARE surface in this study. The material heating method was carried out by covering the surface of the material with a coating spray or an adhesive tape with known emissivity that ranges between 0.9 and 1. GLARE samples were then uniformly heated to a known and steady state temperature level above the ambient temperature, this can be done by using a hot plate, a thermal imaging camera, and surface temperature thermocouples. The spray coating or tape is applied on a part of the surface of the material while the remaining part is kept in the same conditions. The samples were then uniformly heated to a known temperature, the thermocouples were attached on the surface and temperature is also monitored in order to match the used temperature of the hot plate. A variety of thermal imaging cameras were used to find the emissivity by measuring the temperature of the coated or taped surface with the known emissivity and the adjacent surface of GLARE surface, individually. The surface temperature of coated or taped surface was measured and the camera was then moved to the non-coated or taped surface region and the emissivity was reduced until the temperature of GLARE surface matched that of the coated or taped area, the recorded emissivity is the emissivity of GLARE surface. The first test was carried out using an Electrophysic PV320 IR camera with a 20° lens, the camera is capable of providing emissivity resolutions in increments of 0.1 and the data was processed using velocity thermal imaging software.

**Figure 2:**
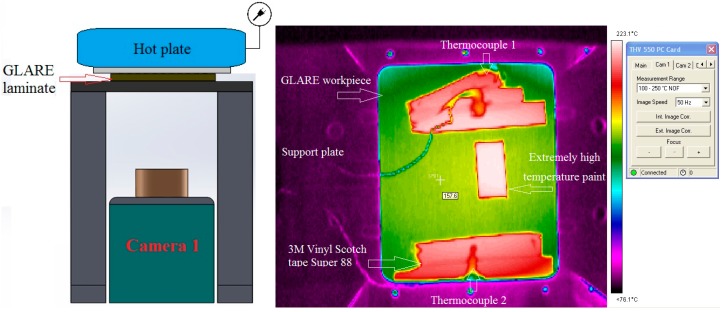
Determination of GLARE emissivity in the second test.

The first and second tests also used K-type high response thermocouples, which were placed in different locations on the upper surface of the laminate to confirm its temperature matches that of the hot plate surface in contact with GLARE laminates. The second and third tests were conducted separately using four calibrated IR cameras, which are capable of detecting emissivity values in the range of 0.01 increments. The second test was carried out in the AMRC-Sheffield, as shown in Figure 2, using two calibrated AGEMA 550 with a 20° lens. 

**Figure 3:**
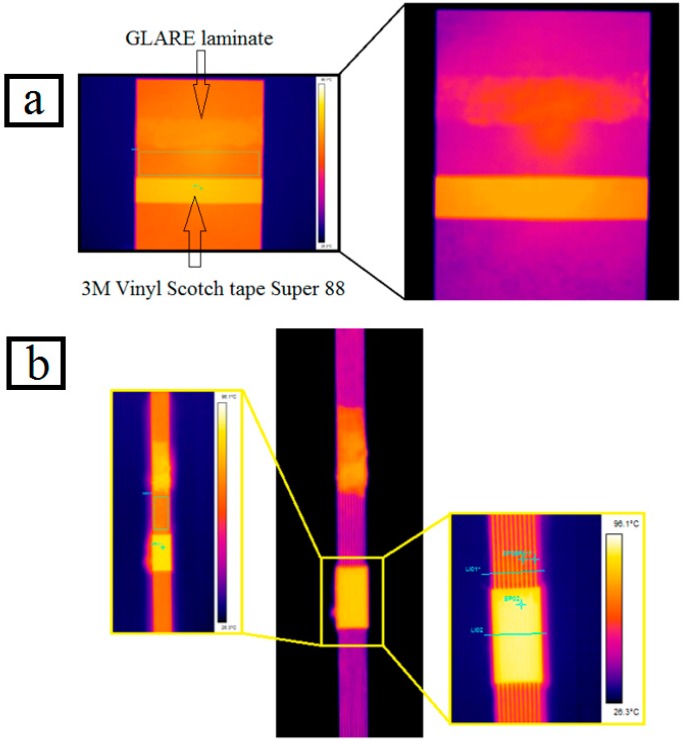
(**a**) Measuring the emissivity of top and bottom surfaces of GLARE laminate in third test. (**b**) Measuring the emissivity of the side of GLARE laminates in the third test.

The third test was conducted by ired.co.uk, a British company specializing in providing thermal imaging services in the U.K, as shown in Figure 3a,b. 

### 2.3. Measuring the Temperature at the Exit Side of Drilled Holes

**Figure 4:**
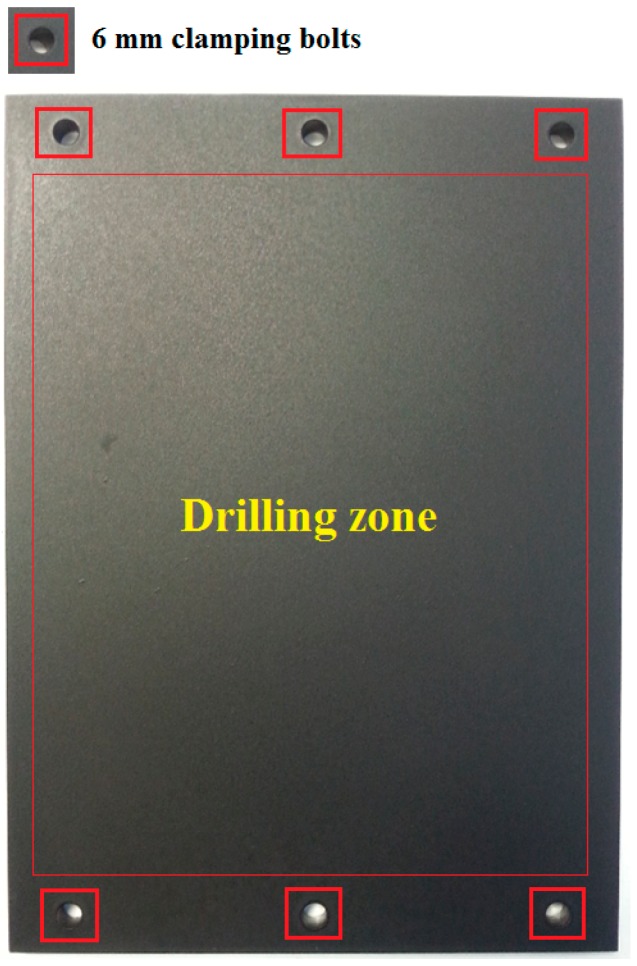
GLARE sample coated with black spray paint.

This part of the study aims to measure the heat-affected zone of the bottom surface of the laminates by determining the maximum temperature during the drilling process. Since the emissivity results of GLARE’s upper and lower surfaces showed that they are not even close to perfect black bodies. In addition, the surface treatment of the upper and lower aluminum sheets in GLARE are highly reflective to light, and since thermal imaging cameras capture the intensity of radiation in the infrared part which is made up of a combination of emitted, transmitted and reflected light. The measured temperature will result from a combination of emitted, transmitted and reflected radiation, which would turn to be inaccurate. Therefore, in order to eliminate the reflectivity of the surfaces, a black spray coating with known emissivity—previously used to determine the emissivity of GLARE—is applied on the lower surface of the samples, as shown in Figure 4. Spraying the back of the plate with a black paint provides a high emissivity value, which is closer to black body definition. This means that the surface will absorb most of the heat and light, and will reflect a minimum amount away from its surface. The commercial name of the paint is E-TECH black extremely high-temperature paint designed for surfaces that are exposed to extremely high temperatures, which provide a high quality finish. The paint is easy to apply with fast curing time and strong adhesion and has a maximum operating temperature rating of 650 °C.

**Figure 5:**
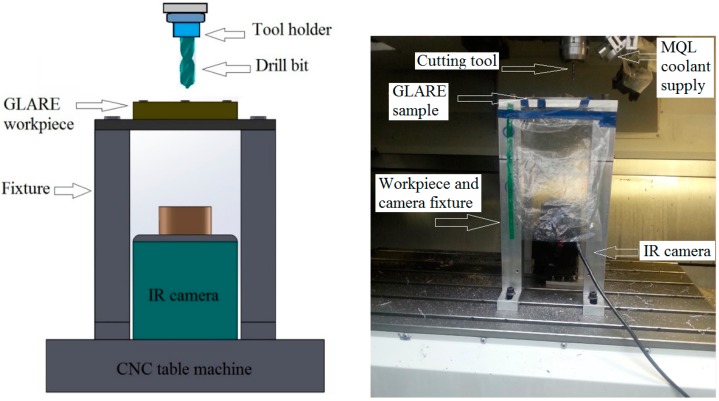
Details of the temperature measurement setup using the IR camera.

The temperature measurement trials were carried out on a MORI SEIKI SV 500 milling machine which has a maximum spindle speed of 10,000 rpm, previously used in our previous works on machining GLARE [6,7,11,66]. Figure 5 shows how the workpiece and camera are set inside the CNC machine. The infrared camera utilized in this study was a calibrated FLIR AGEMA thermovision 550 camera with a built-in 20° lens and 320 × 240 pixels optical resolution. The camera has an operating measuring range of −20 °C to 250 °C and up to 1500 °C with a standard filter. The camera has a spectral range 3.6 to 5 μm and 50 frames per second (fps). The camera is operated using a FLIR tools software, which was also used to process the recorded data from IR camera. The camera was placed vertically with the lenses facing towards the backside surface of the GLARE sample. The fixture was then covered with a thick non-transparent cover to reduce the amount of light entering the chamber to a minimum. 

The drilling parameters (spindle speed and feed rate) used in GLARE 2B and GLARE 3 under dry and MQL conditions are shown in Table 5. For each cutting condition, a set of 10 holes were drilled in a row on the workpiece and temperature was measured continuously at the exit and the highest temperature recorded from the last seven holes was taken as the maximum temperature.

## 3. Results and Discussion

### 3.1. The Effect of Fiber Orientation on Drilling Workpiece Temperature 

**Figure 6:**
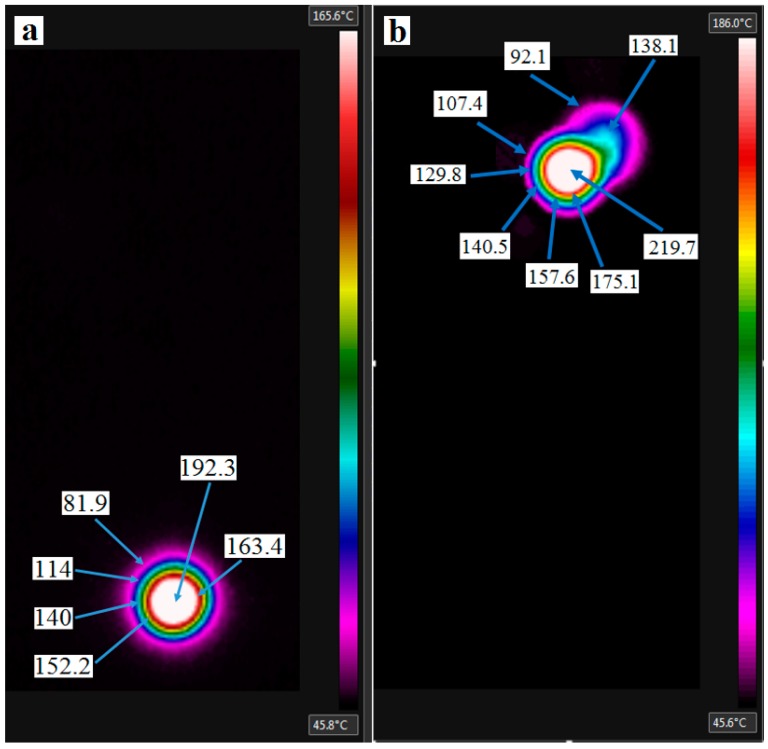
Maximum temperature readings when drilling GLARE 2B 11/10 at (**a**) 6000 rpm and 300 mm/min using MQL; (**b**) 9000 rpm and 900 mm/min showing burr cap separation under dry conditions.

Figure 6 shows the maximum temperature readings of two holes drilled in GLARE 2B 11/10. Figure 6a shows the temperature profiles for the last aluminum sheets just before it separates from the workpiece. Figure 6b shows the maximum temperature profiles of a burr cap separating from the workpiece. The temperature contour profile of the hole in each figure shows that different regions of temperature forms uniformly around the hole periphery during the drilling process. It was found that the temperature decreases when moving away from the hole center (see Figure 6a) the center of the hole, and up to its edges, showed the highest temperature readings with maximum temperature recorded to be around 192.3 °C in this case, and with an average reading of 176.1 °C up to the hole vicinity. Figure 6b shows an aluminum burr cap prior separating from the bottom surface of the hole, which gives an estimation of the temperature of the evacuated chips during the drilling process indicating that it can also reach high-temperature levels similar to those observed on the machined hole surface.

It was observed that the maximum temperature reading decays rapidly, which could be due to the high thermal conductivity of aluminum sheets leading to quick heat dissipation before allowing a sufficient time for heat built-up in the workpiece. The thermal conductivity of S2/FM94 adhesive epoxy system is 1.1–1.4 W/m.K (1.45 W/m.K for S2 glass fibers) while Al2024-T3 alloy is 121 W/m.K [67,68]. Solid carbide drills are around 110 W/m.K, which means that glass fiber layers in GLARE are poor conductors of heat. During the drilling process, the heat accumulates in glass fiber layers and is then conducted to the adjacent aluminum sheets and the cutting tool. The rise of temperature in glass fiber layers can influence the physical properties of the epoxy system, such that the increase in cutting temperatures can soften the epoxy matrix and induce greater instability in the fibers due to micro-buckling [21]. It also indicates that the drilling temperatures could exceed the softening temperature of the epoxy used in GLARE, which has a service temperature (glass transition temperature T_g_) of 103 °C in dry conditions [1]. The rise in cutting temperature weakens the epoxy and affect the bond interface between the glass fiber matrix that plays a significant role in the transfer of stresses in glass fiber layers during the drilling process. The heat generated during the drilling process also contributes to the rapid deterioration of the compressive strength of the fibers. The hole surfaces undergo softening of the matrix material, which causes smearing over the hole boundaries due to the heat generated during the machining process.

Results showed that the drilling temperature increased with the increase of the feed rate. For example, increasing the feed rate from 300 mm/min to 600 mm/min at a constant spindle speed of n= 3000 rpm in GLARE 2B 8/7, the drilling temperatures increased from 163.2 °C to 185.6 °C. Increasing the feed rate to f= 900 mm/min increased the drilling temperatures slightly, reaching 188.1 °C, which could be due to the increase in chip thickness and friction with increasing the feed rate. As feed increases, the chip is thicker and the larger thickness-to-surface area of the chip is cut per revolution, which means there is less opportunity for the heat to be dissipated, hence temperature increases. The finding disagrees with previous studies on drilling monolithic aluminum alloy, CFRP/Al7075-T651 and CFRP/Al6061-T6 stacks [23,34,41,69], which observed that the cutting tool temperature decreased with increase in feed rate for the same spindle speed and drilling depth values due to the increase in material removal rate and, therefore, larger amounts of heat are carried away by the chip, leading to less heat being conducted into the workpiece [69]. However, in another study on drilling titanium alloys, increasing the feed rate increased the drilling temperatures for the same spindle speed and drilling depth [43]. This could be also due to the different cutting parameters and tools used for each study, which could adversely affect the temperature measurement process and results. 

**Figure 7:**
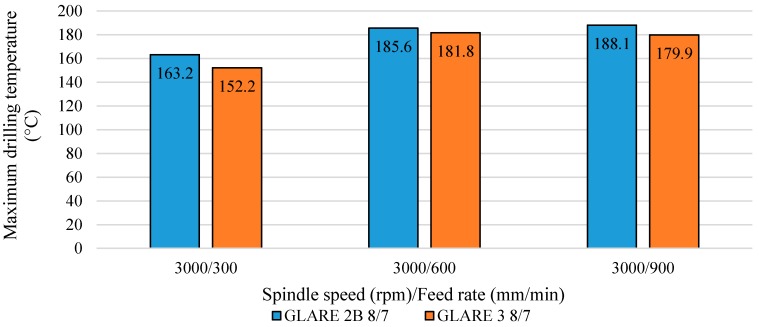
Maximum drilling temperature and the influence of fiber orientation in GLARE.

Figure 7 shows the maximum temperature readings of holes drilled in GLARE 2B and GLARE 3 under the same cutting parameters. The results showed that the maximum drilling temperatures in GLARE 3 8/7 were lower than in GLARE 2B 8/7, which indicates that the fiber orientation can influence the workpiece temperature during the machining process. Zitoune et al. [55] previously reported that the cutting temperature depends on the fiber orientation such that it is higher when drilling with 90° fiber orientation than with 0° due to higher failure stresses. At 90° the fibers are bent then cut by shearing in bulk while at 0°, the chip is formed following a failure in compression. The failure stresses in bending fibers are higher than those in compression and therefore require larger energies (cutting forces) which increase the friction as the tool tip passes through the fibers thus increasing the heating. For example, when drilling at f= 900 mm/min feed rate and n= 3000 rpm spindle speed, the maximum drilling temperature recorded was 188.1 °C in GLARE 2B 8/7 and 179.9 °C in GLARE 3. Fibers oriented at different directions have different values of thermal conductivity exhibited by the matrix and the fiber, which affect the temperature rise during the drilling process. The rise in temperature with the feed rate increase may also be taken accounted for in burr formation, as temperature and thrust force increase, the bottom aluminum sheet experiences an increased ductility, which increases the size of the formed burrs. The results agree with the findings of Ghafarizadeh et al. [51], who previously reported that the fiber orientation has significant effects on the cutting forces and cutting temperature when milling unidirectional CFRPs, such that the maximum and minimum values are obtained at fiber orientations of 90° and 0°, respectively. The difference in drilling temperatures did not exceed 10 °C in the tested cutting parameters, which could be due to the small thickness of glass fiber layers in GLARE. As reported in one of our previous studies, the cutting force results showed a minor difference in thrust force between GLARE 2B and GLARE 3, arising from the difference in fiber orientation for the same thickness [7]. However, it was observed that the torque in GLARE 2B was higher than in GLARE 3 at same tested cutting parameters, which could have led to higher frictions between the cutting tool and the workpiece, causing a greater temperature rise in GLARE 2B. In addition, the relatively small thickness of the fiber layers might not be enough to promote a significant temperature rise due to difference in fiber orientation. The recorded temperatures are within the range obtained in previous studies on drilling aluminum alloys, such as Al2024-T3, Al6061-T6 and Al7075-T6, which showed that maximum temperatures ranged between 131 °C and 360 °C when drilling monolithic aluminum, and up to 199 °C in composite metal stacks [23,34,40,41,43,50]. 

### 3.2. The Effect of MQL Coolant on Workpiece Temperature

**Figure 8:**
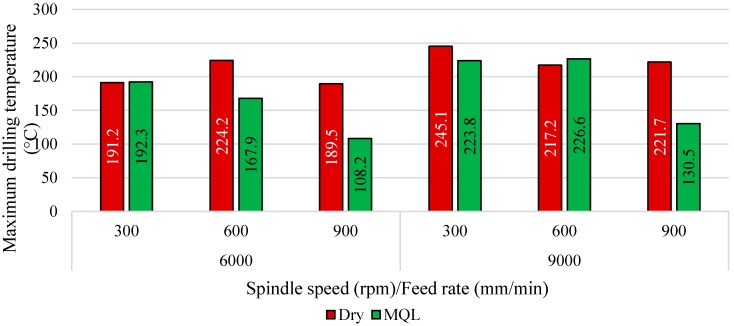
Comparison of the exit temperature of holes drilling under MQL and dry conditions.

Figure 8 shows a comparison of hole temperature at exit between dry and MQL tests. The MQL tests were carried out using 20 mL/h flow rate coolant and an air pressure of 3 bars since they proved to give best results among all other tested flow rates and air pressures in one of our previous studies. Results showed that the application of MQL coolant can reduce machining temperatures when drilling at high feed rates of f= 900 mm/min. This could be due to the lubrication effect, which reduces friction around hole walls during drill-workpiece contact. Drilling at feed rates of f= 300 mm/min and spindle speeds of n= 6000 rpm gave similar results to those obtained under dry cutting, which could be due to rapid evaporation of lubricant before having sufficient time to lubricate the cutting tool and workpiece. Under the same feed rate but at higher spindle speeds of n= 9000 rpm, the application of MQL reduced the temperature by approximately 22 °C, and increased by approximately 10 °C when drilling at feed rate of f= 600 mm/min, which indicate that the combination of spindle speed and feed rate plays a significant role in developed temperature in GLARE. When drilling GLARE using spindle speeds of n= 6000 and 9000 rpm, increasing the feed rate tended to decrease the cutting temperature which indicates that MQL becomes more effective when the drilling time is reduced (i.e., when the feed rate increases). This could be due to the small amounts of lubricant used in MQL, which tend to evaporate rapidly, thus, limiting its ability to lubricate the cutting tool-workpiece properly. 

**Figure 9:**
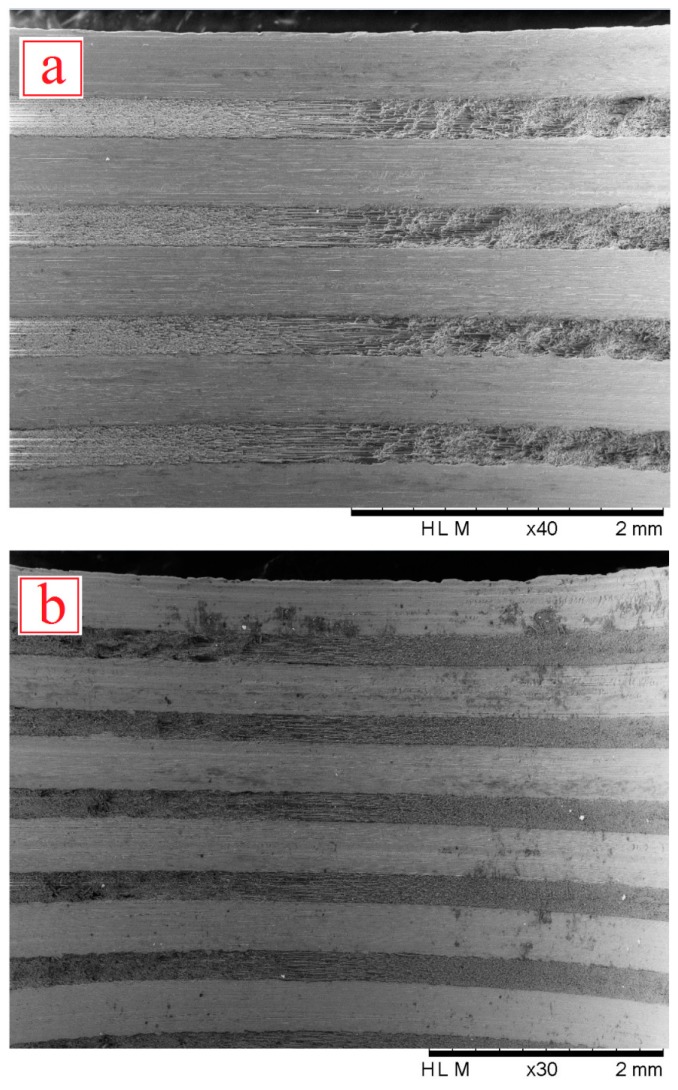
SEM images of the upper borehole surface condition of holes under (**a**) MQL and (**b**) dry.

The use of MQL cooling was found to reduce the amount of waste formed on the machined surfaces of the laminate, especially when drilling at high spindle speeds and low feed rates, as shown in Figure 9. The MQL lubricant reduces the friction between the workpiece and cutting tool while the air pressure assists in transporting the chips and waste material away from the cutting zone. Increasing the spindle speed increased the workpiece temperature due to increased rubbing between the cutting tool and the borehole surface per unit of time, which causes higher friction, thus raising the machining temperatures. Increasing the feed rate showed somewhat different results depending on the spindle speed used. The reduction in cutting temperatures with the increase of the feed rate is due to reduced tool-workpiece contact time. Although no comparison was made to determine the impact of laminate thickness on developed temperatures, it is speculated that a larger laminate thickness gives a larger contact area that produces more heat, while chip evacuation in thinner laminates is easier and, therefore, the heat produced is lower due to smaller contact area, which also reduced the chip adherence to the cutting tool [1]. At higher spindle speeds, a higher temperature is generated, which increases the tendency of chip and fibers to adhere to the cutting tool and then be forced into the laminate edges and machined surfaces and is known as a waste material causing delamination and deformations [1].

The use of MQL cooling was found to reduce the amount of waste formed on the machined surfaces of the laminate, especially when drilling at high spindle speeds and low feed rates, as shown in Figure 9. The MQL lubricant reduces the friction between the workpiece and cutting tool while the air pressure assists in transporting the chips and waste material away from the cutting zone. Increasing the spindle speed increased the workpiece temperature due to increased rubbing between the cutting tool and the borehole surface per unit of time, which causes higher friction, thus raising the machining temperatures. Increasing the feed rate showed somewhat different results depending on the spindle speed used. The reduction in cutting temperatures with the increase of the feed rate is due to reduced tool-workpiece contact time. Although no comparison was made to determine the impact of laminate thickness on developed temperatures, it is speculated that a larger laminate thickness gives a larger contact area that produces more heat, while chip evacuation in thinner laminates is easier and, therefore, the heat produced is lower due to smaller contact area, which also reduced the chip adherence to the cutting tool [1]. At higher spindle speeds, a higher temperature is generated, which increases the tendency of chip and fibers to adhere to the cutting tool and then be forced into the laminate edges and machined surfaces and is known as a waste material causing delamination and deformations [1].

## 4. Conclusions 

The drilling process of GLARE fiber metal laminates was experimentally evaluated using non-contact infrared techniques to determine the impact of cutting parameters (spindle speed and feed rate), fiber orientation and the use of coolants on the maximum developed temperature at the bottom surface of the machined hole in GLARE laminates. The following results can be concluded:

- The temperature of the bottom surface of the workpiece increased as drilling progress. The investigation indicated that both drilling parameters, i.e., spindle speed and feed rate, influence the cutting temperature during machining.
- The maximum temperature at the bottom surface when drilling GLARE fiber metal laminates was measured by employing infrared temperature techniques. The measured temperature reached up to 245.1 °C at a spindle speed of n= 9000 rpm and a feed rate of f= 300 mm/min when drilling at room temperature and up to 192.3 °C when drilling under minimum quantity lubrication cooling.
- In GLARE 2B 8/7 and GLARE 3 8/7, the maximum temperature was found to increase with the increase of the feed rate when drilling at spindle speed n= 3000 rpm under dry conditions (room temperature). Increasing the feed rate from f= 300 mm/min to f= 900 mm/min increased the temperature by 15.2% in GLARE 2B 8/7 and by 18.2% in GLARE 3 8/7.
- The difference in maximum drilling temperature between GLARE 2B and GLARE 3 ranged from 4 to 10 °C under the tested cutting parameters.
- The fiber orientation in GLARE laminates influences the maximum drilling temperature, such that GLARE laminates with same fiber orientation as GLARE 2B (90°/90°) will be susceptible to higher drilling temperatures than laminates with different fiber orientation, such as GLARE 3 (0°/90°).
- In GLARE 2B 11/10, the maximum temperature was found to depend on the level of the feed rate when drilling at spindle speed n= 6000 and 9000 rpm under dry (room temperature) and MQL conditions, increasing the feed rate from f= 300 mm/min to f= 600 mm/min increased the temperature by 17.2% under dry and decreased it by 14.5% using MQL.
- The application of MQL cooling can considerably reduce the machining temperatures in GLARE, the efficiency of MQL increase with the increase of the feed rate due to improved lubrication and reduced drilling time.
- The largest reduction in workpiece temperature when using MQL compared to dry drilling was achieved when drilling at spindle speed n= 6000 and 9000 rpm and feed rates of f= 900 mm/min. The reduction in maximum drilling temperature reached 75%.
- The infrared thermos vision techniques used in the current study suggests the possibility of using those methods to measure the temperatures fields in the machining processes, which can be also used as an effective tool for temperature monitoring. The emissivity of GLARE found in the current study can be used as an input for future studies for the purpose of monitoring the machining temperature of GLARE laminates.

## Figures and Tables

**Table 1:** Summary of the reported studies on machining fiber metal laminates [11].

Machining process	Cutting Tools Used	Workpiece Description	Objectives	Ref
AWJM	Orifice: Sapphire, diameter 0.381 mm Nozzle Length 76.2 mm, diameter 1.016 mm, Grit type Garnet, Mesh 80 Impact angle 90°	Titanium/Graphite (Ti/Gr)	Mrr K	[5]
	Diamond orifice, diameter of 0.3 mm Insert diameter of 1.1 mm, length 51 mm and 90° impact angle, Garnet: olivine abrasive, Mesh 60	GLARE 2–3/2–0.2 GLARE 2–3/2–0.3 GLARE 4–5/4–0.3	B D Ke	[4]
Milling	Not reported	GLARE	D F M	[3]
Drilling	5 mm HSS TiN coated drill, HSS with 8% Co drill, carbide tipped HSS drill 5 and 5.5 mm solid carbide drill 4.8 mm diamond tipped HSS drill	GLARE 3–3/2–0.3 GLARE 3–2/1–0.3 GLARE3–4/3–0.3	B C Z	[9]
	6 mm uncoated carbide drill	GLARE-like made of Al2024 sheets and fiberglass type R	C Z	[8]
	6.35 solid carbide drills	GLARE 5 3/2–0.3 GLARE 6 3/2–0.3	C Z F D B Ae	[10]
	6 mm TiAlN coated carbide drill	GLARE 2B 11/10–0.4 GLARE 2B 8/7–0.4 GLARE 3 8/7–0.4	C R Q B Z Y O	[6,7,11,12]

Symbols➜ C: Cutting forces, D: Delamination, M: Cutting mechanisms R: surface roughness, B: Burr formation, W: Tool wear, Z: Hole Size, T: Drilling Temperature, Y: Circularity, H: Hardness, F: Chip formation, A: Acoustic emission, Q: Stacking sequence, O: Coolants, Ae: Absolute energy. AWM: Abrasive Water Jet Machining K: Kerf quality Mrr: Material removal rate Ke: Kerf taper.

**Table 2:** GLARE grades used in the drilling experiments and their properties [6,7,11].

Material	Glare 3 8/7-0.4	Glare 2B 8/7-0.4	Glare 2B 11/10-0.4
Thickness of Aluminum layer (mm)	0.4064	0.4064	0.4064
Thickness of S2 glass fiber layer (mm)	0.266	0.266	0.266
Total thickness (mm)	5.113	5.113	7.130
Metal Volume Fraction % (M.V.F.)	63.58%	63.58%	62.69%
Workpiece dimensions (mm)	200 × 150	200 × 150	200 × 150

**Table 3:** Mechanical Properties of S2-Glass fiber prepreg and Al2024-T3 [11,62,63,64,65].

Mechanical Property		UD S2 Glass/FM 94 Epoxy Prepreg $V_{F}=60\%$	Al2024-T3	Units
Young Modulus (E)	L	54–55	72.2	GPa
	T	9.4–9.5	-	
Ultimate tensile strength (σ)	L	2640	455	MPa
	T	57	448	
Ultimate strain % (ε)	L	3.5–4.7	19	-
	T	0.6	-	
Shear Modulus (G)	L	5.55	27.6	GPa
	T	3	-	
Poisson’s ratio (ν)	L	0.33	0.33	-
	T	0.0575	-	
Density (ρ)	-	1980	2770	kg/m^3^
Thermal expansion coefficient (α)	L	3.9–6.1	23.4	(1/°C) 10^−6^
	T	26.2–55.2	23.4	
Thermal conductivity (K)	L	1.1–1.4	121	W/m-K
	T	0.43–0.53	-	

The symbols L and T stand for longitudinal (the rolling direction for the metal) and transverse directions, respectively.

**Table 4:** Emissivity of GLARE fiber metal laminates using three IR cameras and thermocouple techniques.

Description	Test 1	Test 2	Test 3
Camera type	Electrophysic PV320 20° lens	AGEMA 550 20° lens	FLIR SC640: 0.4 m away, 24° lens FLIR B400: 0.1 m away, 45° lens
No. of cameras used	1	2	2
Type of coating/tape applied	Black spray paint	Extremely high-temperature paint & 3M Vinyl Scotch tape Super 88	3M Vinyl Scotch tape Super 88
Emissivity of coating used	0.94	0.94 & 0.95	0.95
Heat source used	Hotplate	Hotplate	Oven
Temperature level used	60–70 °C	60–90 °C	90°C
T ambient	24 °C	24 °C	24 °C
T reflect	-	21 °C	21 °C
Thermocouples used	Yes	Yes	No
E1 (Top and Bottom)	0.8	0.82	0.832–0.843
E2 (S2-FM94 plies)	0.6	0.6	0.594–0.683
E3 (Al2024 sheets)	0.4	-	0.276–0.395
Distance between camera and target surface	0.5 m	0.45 m	0.4 m and 0.1 m

**Table 5:** Cutting parameters used in drilling temperature measurements in dry and MQL trials.

	Spindle Speed (rpm)		
Feed rate (mm/min)	3000	6000	9000
300	A,B	C,D	C,D
600	A,B	C,D	C,D
900	A,B	C,D	C,D

A: G2B 8/7 B: G3 8/7 C: G2B 11/10 D: G2B 11/10 MQL.

## References

[1] Vlot A., Gunnink J.W. (2001). Fibre Metal Laminates: An Introduction.

[2] Sinke J. (2003). Manufacturing of glare parts and structures. Appl. Compos. Mater..

[3] Brinksmeier E. (2014). Machinability of carbon-fiber-reinforced and glare materials. CIRP Encycl. Prod. Eng..

[4] Paul S., Hoogstrate A., Van Praag R. (2002). Abrasive water jet machining of glass fibre metal laminates. Proc. Inst. Mech. Eng. Part B J. Eng. Manuf..

[5] Ramulu M., Pahuja R., Hashish M., Isvilonanda V. (2015). Abrasive waterjet machining effects on kerf quality in thin fiber metal laminate. 2015 WJTA-IMCA Conference and Expo.

[6] Giasin K., Ayvar-Soberanis S., Hodzic A. (2016). The effects of minimum quantity lubrication and cryogenic liquid nitrogen cooling on drilled hole quality in glare fibre metal laminates. Mater. Des..

[7] Giasin K., Ayvar-Soberanis S., Hodzic A. (2015). An experimental study on drilling of unidirectional glare fibre metal laminates. Compos. Struct..

[8] Tyczynski P., Lemanczyk J., Ostrowski R. (2014). Drilling of CFRP, GFRP, glare type composites. Aircr. Eng. Aerosp. Technol..

[9] Coesel J.F.W. (1994). Drilling of Fibre-Metal Laminates. Master Thesis.

[10] Pawar O.A., Gaikhe Y.S., Tewari A., Sundaram R., Joshi S.S. (2015). Analysis of hole quality in drilling glare fiber metal laminates. Compos. Struct..

[11] Giasin K., Ayvar-Soberanis S., Hodzic A. (2016). Evaluation of cryogenic cooling and minimum quantity lubrication effects on machining glare laminates using design of experiments. J. Clean. Prod..

[12] Giasin K., Ayvar-Soberanis S., French T., Phadnis V. (2016). 3d finite element modelling of cutting forces in drilling fibre metal laminates and experimental hole quality analysis. Appl. Compos. Mater..

[13] Groover M.P. (2007). Fundamentals of Modern Manufacturing: Materials Processes, and Systems.

[14] Parashar B.N., Mittal R. (2002). Elements of Manufacturing Processes.

[15] Rawat S., Attia H. (2009). Wear mechanisms and tool life management of WC–CO drills during dry high speed drilling of woven carbon fibre composites. Wear.

[16] El-Hofy H.A.-G. (2013). Fundamentals of Machining Processes: Conventional and Nonconventional Processes.

[17] Ozek C., Demir Z. (2013). Investigate the friction drilling of aluminium alloys according to the thermal conductivity. Tem J. Technol. Educ. Manag. Inf..

[18] Sheikh-Ahmad J.Y. (2009). Machining of Polymer Composites.

[19] Davim J.P. (2012). Tribology in Manufacturing Technology.

[20] DeGarmo E.P., Black J.T., Kohser R.A. (2003). Materials and Processes in Manufacturing.

[21] Khan Z.M. (1991). A Study of the Drilling of Advanced Carbon Fibre Composites. Ph.D. Thesis.

[22] Fleischer J., Pabst R., Kelemen S. (2007). Heat flow simulation for dry machining of power train castings. CIRP Ann. - Manuf. Technol..

[23] Wang C.-Y., Chen Y.-H., An Q.-L., Cai X.-J., Ming W.-W., Chen M. (2015). Drilling temperature and hole quality in drilling of CFRP/aluminum stacks using diamond coated drill. Int. J. Precis. Eng. Manuf..

[24] Taskesen A., Kutukde K. (2015). Non-contact measurement and multi-objective analysis of drilling temperature when drilling B_4_C reinforced aluminum composites. Trans. Nonferrous Metals Soc. China.

[25] Shetty R., Pai R., Kamath V., Rao S.S. (2008). Steam as coolant and lubricant in turning of metal matrix composites. J. Zhejiang Univ. Sci. A.

[26] Kolesnyk V., Zajac J., Radchenko S., Adamian M. (2015). The effect of cutting temperature on hole quality when drilling CFRP/metal stack. Вісник НТУ.

[27] Kaynak Y. (2014). Evaluation of machining performance in cryogenic machining of inconel 718 and comparison with dry and mql machining. Int. J. Adv. Manuf. Technol..

[28] Manimaran G., Venkatasamy R. (2014). Influence of cryogenic cooling on surface grinding of stainless steel 316. Cryogenics.

[29] Dhar N.R., Ahmed M.T., Islam S. (2007). An experimental investigation on effect of minimum quantity lubrication in machining AISI 1040 steel. Int. J. Mach. Tools Manuf..

[30] Abhang L., Hameedullah M. (2010). Chip-tool interface temperature prediction model for turning process. Int. J. Eng. Sci. Technol..

[31] Kitagawa T., Kubo A., Maekawa K. (1997). Temperature and wear of cutting tools in high-speed machining of inconel 718 and Ti-6Al-6V-2Sn. Wear.

[32] Nedić B.P., Erić M.D. (2014). Cutting temperature measurement and material machinability. Therm. Sci..

[33] Bono M., Ni J. (2002). A method for measuring the temperature distribution along the cutting edges of a drill. J. Manuf. Sci. Eng..

[34] Bağci E., Ozcelik B. (2006). Investigation of the effect of drilling conditions on the twist drill temperature during step-by-step and continuous dry drilling. Mater. Des..

[35] El-Wardany T.I., Mohammed E., Elbestawi M.A. (1996). Cutting temperature of ceramic tools in high speed machining of difficult-to-cut materials. Int. J. Mach. Tools Manuf..

[36] Ueda T., Sato M., Hosokawa A., Ozawa M. (2008). Development of infrared radiation pyrometer with optical fibers—two-color pyrometer with non-contact fiber coupler. CIRP Ann.-Manuf. Technol..

[37] Sato M., Aoki T., Tanaka H., Takeda S. (2013). Variation of temperature at the bottom surface of a hole during drilling and its effect on tool wear. Int. J. Mach. Tools Manuf..

[38] Ueda T., Nozaki R., Hosokawa A. (2007). Temperature measurement of cutting edge in drilling -effect of oil mist. CIRP Ann. - Manuf. Technol..

[39] Aramcharoen A., Chuan S.K. (2014). An experimental investigation on cryogenic milling of inconel 718 and its sustainability assessment. Procedia CIRP.

[40] Rivero A., Aramendi G., Herranz S., de Lacalle L.L. (2006). An experimental investigation of the effect of coatings and cutting parameters on the dry drilling performance of aluminium alloys. Int. J. Adv. Manuf. Technol..

[41] Dandekar C., Orady E., Mallick P. (2007). Drilling characteristics of an e-glass fabric-reinforced polypropylene composite and an aluminum alloy: A comparative study. J. Manuf. Sci. Eng..

[42] Kalidas S., DeVor R.E., Kapoor S.G. (2001). Experimental investigation of the effect of drill coatings on hole quality under dry and wet drilling conditions. Surf. Coat. Technol..

[43] Le Coz G., Marinescu M., Devillez A., Dudzinski D., Velnom L. (2012). Measuring temperature of rotating cutting tools: Application to MQL drilling and dry milling of aerospace alloys. Appl. Thermal Eng..

[44] Vernaza-Pena K., Mason J., Li M. (2002). Experimental study of the temperature field generated during orthogonal machining of an aluminum alloy. Exp. Mech..

[45] Li R., Shih A.J. (2007). Tool temperature in titanium drilling. Ann. Arbor.

[46] Li R., Shih A.J. (2007). Spiral point drill temperature and stress in high-throughput drilling of titanium. Int. J. Mach. Tools Manuf..

[47] Fang F., Lee L., Liu X. (2005). Mean flank temperature measurement in high speed dry cutting of magnesium alloy. J. Mater. Process. Technol..

[48] Weinert K., Kempmann C. (2004). Cutting temperatures and their effects on the machining behaviour in drilling reinforced plastic composites. Adv. Eng. Mater..

[49] Krishna A.S.R., Reddy P.R. (2012). Temperature prediction in orthogonal machining of A1/SICP composites. Int. J. Emerg. Technol. Adv. Eng..

[50] Brinksmeier E., Fangmann S., Rentsch R. (2011). Drilling of composites and resulting surface integrity. CIRP Ann. - Manuf. Technol..

[51] Ghafarizadeh S., Lebrun G., Chatelain J.-F. (2015). Experimental investigation of the cutting temperature and surface quality during milling of unidirectional carbon fiber reinforced plastic. J. Compos. Mater..

[52] Merino-Pérez J., Royer R., Ayvar-Soberanis S., Merson E., Hodzic A. (2015). On the temperatures developed in CFRP drilling using uncoated WC-CO tools part I: Workpiece constituents, cutting speed and heat dissipation. Compos. Struct..

[53] Takeyama H., Iijima N. (1988). Machinability of glassfiber reinforced plastics and application of ultrasonic machining. CIRP Ann. - Manuf. Technol..

[54] Sreejith P., Krishnamurthy R., Malhotra S., Narayanasamy K. (2000). Evaluation of pcd tool performance during machining of carbon/phenolic ablative composites. J. Mater. Process. Technol..

[55] Zitoune R., Collombet F., Lachaud F., Piquet R., Pasquet P. (2005). Experiment–calculation comparison of the cutting conditions representative of the long fiber composite drilling phase. Compos. Sci. Technol..

[56] Abhishek K., Datta S., Mahapatra S.S. (2013). Response surface modeling on machining of CFRP composites: Effect of process variables on surface roughness, MRR and tool-tip temperature. Int. J. Mechan. Eng. Res..

[57] Haddad M., Zitoune R., Bougherara H., Eyma F., Castanié B. (2014). Study of trimming damages of CFRP structures in function of the machining processes and their impact on the mechanical behavior. Compos. Part B Eng..

[58] Saleem M., Toubal L., Zitoune R., Bougherara H. (2013). Investigating the effect of machining processes on the mechanical behavior of composite plates with circular holes. Compos. Part A Appl. Sci. Manuf..

[59] Haddad M., Zitoune R., Eyma F., Castanie B. (2014). Study of the surface defects and dust generated during trimming of CFRP: Influence of tool geometry, machining parameters and cutting speed range. Compos. Part A Appl. Sci. Manuf..

[60] Wang B., Gao H., Cao B., Zhuang Y., Zhao Z. (2014). Mechanism of damage generation during drilling of carbon/epoxy composites and titanium alloy stacks. Proc. Inst. Mech. Eng. Part B J. Eng. Manuf..

[61] Alderliesten R.C. (2005). Fatigue Crack Propagation and Delamination Growth in Glare.

[62] De Vries T.J. (2001). Blunt and Sharp Notch Behaviour of Glare Laminates.

[63] Mohit G., Frank A., Michael F., Galib A. (2011). Predicting bearing strength of fiber metal laminates via progressive failure analysis. 52nd aiaa/asme/asce/ahs/asc Structures, Structural Dynamics and Materials Conference.

[64] Yaghoubi A.S., Liaw B. (2013). Damage assessments of ballistic impact behaviors of glare 5 (3/2) beams with various stacking sequences. Dynamic Behavior of Materials.

[65] Hagenbeek M. (2005). Characterisation of Fibre Metal Laminates under Thermomechanical Loadings.

[66] Giasin K., Hodzic A., Phadnis V., Ayvar-Soberanis S. (2016). Assessment of cutting forces and hole quality in drilling Al2024 aluminium alloy: Experimental and finite element study. Int. J. Adv. Manuf. Technol..

[67] De Vries T.J. (2001). Blunt and Sharp Notch Behaviour of Glare Laminates.

[68] Seo H. (2008). Damage Tolerance and Durability of Glare Laminates.

[69] O’Sullivan D., Cotterell M. (2002). Workpiece temperature measurement in machining. Proc. Inst. Mech. Eng. Part B J. Eng. Manuf..

